# Evaluating the Applicability of the Self-Assessment Tool for Family Caregivers (SSA-PA) in Care Counseling According to Section 45 of the German Social Code (SGB XI): A Mixed-Methods Study

**DOI:** 10.3390/healthcare14050577

**Published:** 2026-02-25

**Authors:** Laura Schwedler, Thomas Ostermann, Jan Ehlers, Gregor Hohenberg

**Affiliations:** 1 Stabsstelle für Digitalisierung und Wissensmanagement, Hochschule Hamm-Lippstadt, 59063 Hamm, Germany; gregor.hohenberg@hshl.de; 2 Fakultät für Gesundheit, Universität Witten-Herdecke, 58455 Witten, Germany; thomas.ostermann@uni-wh.de (T.O.); jan.ehlers@uni-wh.de (J.E.)

**Keywords:** care advice, self-assessment, family caregivers, coping with stress, qualitative study, feasibility study

## Abstract

**Background/Objectives**: Family caregivers play a central role in long-term care but are frequently exposed to considerable physical, emotional, and social strain. In Germany, care counseling pursuant to §45 SGB XI aims to identify caregiver burden at an early stage and provide preventive, resource-oriented support. Structured self-assessment tools may facilitate reflective dialogue within time-limited counseling sessions. The Self-Assessment Tool for Family Caregivers (SSA-PA) was developed to support this process; however, empirical evidence regarding its applicability in statutory counseling settings remains limited. This exploratory mixed-methods study aimed to generate empirical insights into (1) the perceived usefulness and acceptance of the SSA-PA among care advisors, (2) opportunities and challenges associated with its practical implementation, and (3) its perceived integration potential within routine counseling practice. **Methods**: Thirteen care advisors working under §45 SGB XI applied the SSA-PA in routine counseling and subsequently completed a structured online survey combining Likert-scale items and open-ended questions. Quantitative data were analyzed descriptively using IBM SPSS Statistics (Version 29), and qualitative responses were examined using thematic analysis. Given the moderate sample size (*n* = 13), analyses were primarily descriptive and exploratory in nature. **Results**: Care advisors reported high perceived usefulness and broad acceptance of the SSA-PA as a structuring and reflective instrument in counseling sessions. The tool was described as supportive in facilitating discussion of caregiver burden across multiple life domains and enhancing transparency of stress-related issues. At the same time, participants identified practical challenges, including time constraints, emotional strain for caregivers, technical barriers, and the need for clearer evaluation outputs. Suggestions for further development included automated result processing, individualized recommendations, and longitudinal tracking functions. **Conclusions**: From the perspective of participating care advisors, the SSA-PA demonstrates promising feasibility and acceptance within statutory preventive counseling under §45 SGB XI. While the findings provide practice-based evidence for its applicability, conclusions regarding effectiveness or outcome improvements cannot be drawn. Further research with larger samples and outcome-oriented designs is required to evaluate its impact on caregiver burden and counseling processes.

## 1. Introduction

Family caregivers constitute a central pillar of long-term care systems worldwide. They provide substantial informal support across physical, emotional, and organizational domains and frequently assume complex care responsibilities over extended periods. While caregiving can be experienced as meaningful, it is consistently associated with elevated risks of physical exhaustion, psychological strain, social isolation, and long-term health impairments [1,2,3,4,5,6,7,8,9]. Early identification of caregiver burden and timely preventive support are therefore considered essential components of sustainable care systems.

In Germany, care counseling pursuant to §45 SGB XI (German Social Code) aims to strengthen caregivers’ resources, promote preventive strategies, and identify stress-related risks at an early stage [10,11,12,13,14,15]. Unlike clinically oriented interventions, counseling under §45 SGB XI is typically time-limited, resource-focused, and embedded in statutory advisory structures. Within this framework, care advisors must efficiently assess caregiver needs, structure complex information, and facilitate reflective dialogue—often within a single session.

However, caregivers frequently underestimate their own burden or focus primarily on the care recipient’s needs rather than their own well-being. This dynamic may delay support-seeking behavior and reduce the preventive potential of counseling services. Structured self-assessment tools represent a low-threshold strategy to enhance self-reflection, support awareness of stress-related domains, and provide a systematic basis for dialogue between caregivers and professionals [16,17,18,19,20,21,22,23,24,25]. International research suggests that such tools can improve communication quality and promote resource-oriented counseling processes. Nevertheless, most existing instruments are either clinically diagnostic, disease-specific, or developed for research settings rather than for statutory preventive counseling contexts.

To address this gap, the Self-Assessment Tool for Family Caregivers (SSA-PA) was developed as a structured, resource-oriented reflection instrument tailored to the German counseling framework under §45 SGB XI [26]. The tool is not intended as a diagnostic instrument but as a facilitative aid that structures discussion across multiple life domains, including physical well-being, emotional strain, social support, and coping strategies.

While the initial development and validation of the SSA-PA have been reported previously [26], empirical evidence regarding its real-world applicability in routine counseling practice remains limited. In particular, the perspective of care advisors who operationalize the tool within statutory and time-constrained settings has not yet been systematically examined.

Therefore, this exploratory mixed-methods study aims to generate empirical insights into the practical implementation of the SSA-PA in care counseling pursuant to §45 SGB XI. Specifically, the study pursues three objectives:

1. To examine the perceived usefulness and acceptance of the SSA-PA from the perspective of care advisors after practical application in routine counseling. This includes assessing whether the tool is considered supportive for structuring consultations and facilitating caregiver self-reflection.
2. To identify opportunities and challenges associated with implementation, including structural, emotional, technical, and organizational barriers that may influence feasibility within time-limited counseling contexts.
3. To explore the perceived integration potential and developmental needs of the SSA-PA, including suggestions for refinement, longitudinal use, and potential transferability to other counseling frameworks beyond the German statutory context.

While the study does not assess effectiveness outcomes, it provides practice-based evidence regarding feasibility, acceptance, and implementation conditions to inform further development and larger-scale evaluations.

## 2. Materials and Methods

### 2.1. Description of the Self-Assessment Tool (SSA-PA)

The Self-Assessment Tool for Family Caregivers (SSA-PA) was developed as a structured, resource-oriented reflection instrument to support family caregivers in systematically assessing stress-related domains in their daily caregiving context. The instrument comprises 23 items across 10 thematic categories, covering physical well-being, emotional strain, social support, coping strategies, and self-care. It includes Likert-scale items, single-choice questions, and open-ended responses to allow both structured assessment and individualized reflection.

The development process, theoretical foundation, and initial psychometric evaluation of the SSA-PA have been described previously [26]. The present study does not re-evaluate the psychometric properties of the tool but focuses on its practical applicability, acceptance, and feasibility within statutory care counseling under §45 SGB XI.

### 2.2. Study Design

This study employed a mixed-methods design, combining quantitative and qualitative approaches to assess the practical use and perceived usefulness of the SSA-PA. Data were collected via an online survey administered to care advisors after they had applied the tool in their counseling practice. The survey aimed to capture:

- Perceptions of usefulness and relevance,
- Acceptance and challenges,
- Suggested improvements for integration into routine counseling processes.

**Figure 1:**
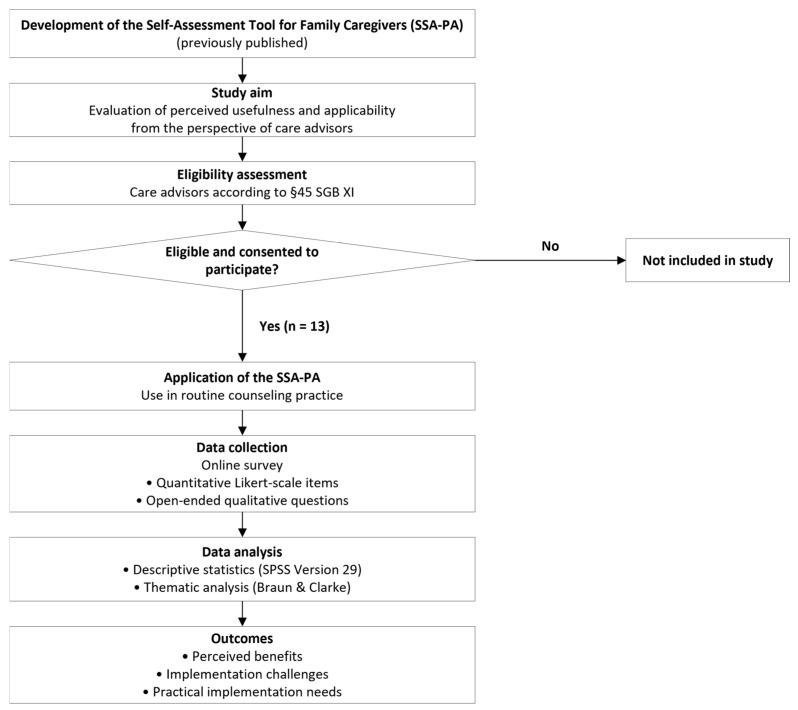
Study flow of the mixed-methods evaluation of the SSA-PA (*n* = 13).

The overall process, including recruitment, tool application, survey administration, and data analysis, is illustrated in Figure 1. The flowchart provides a concise overview of the study phases, allowing readers to understand the sequence of actions and the integration of both quantitative and qualitative components. The overall study process, including eligibility assessment, implementation phase, and mixed-methods evaluation, is illustrated in Figure 1.

### 2.3. Participants and Recruitment

The target group consisted of active care advisors working in accordance with §45 SGB XI who regularly counsel family caregivers.

Inclusion criteria:

- Active professional role as care advisor under §45 SGB XI,
- Direct counseling of family caregivers,
- Willingness to apply the SSA-PA in practice,
- Completion of the evaluation survey.

No additional exclusion criteria were defined to reflect real-world counseling diversity.

A total of 13 care advisors participated in the study. Recruitment was conducted via professional networks and institutional contacts. Participation was voluntary and anonymous.

Sociodemographic Categorization:

Age groups were categorized into intervals (e.g., <30, 30–39, 40–49, 50–59, ≥60 years) based on commonly used demographic classifications in workforce statistics to ensure comparability while maintaining anonymity in a relatively small sample. The grouping also allowed for meaningful descriptive analysis without compromising participant confidentiality.

Sociodemographic characteristics are presented in Table 1.

### 2.4. Development of the Survey

The survey was developed to assess the perceived usefulness, acceptance, feasibility, and developmental needs related to the SSA-PA.

Item development was informed by:

1. Caregiver burden: Physical, emotional, and social stressors relevant for family caregivers [1,2,3,4,5,6,7,8,9,16,17,18,19,20,21,22,23,24,25,27,28,29,30,31,32,33].
2. Care counseling in accordance with §45 SGB XI: Practical guidance on identifying stressors, promoting resources, and supporting caregivers [10,11,12,13,14,15,34].
3. Methodological standards for self-assessment tools: Question clarity, relevance, and format for capturing both quantitative and qualitative data [9,12,16,17,18,25].
4. International research: Key stress domains and caregiver experiences from different countries to ensure items were comprehensive and relevant [1,2,3,4,20,22,28].

The resulting instrument comprised 23 items across 10 thematic categories (see Table 2), combining Likert-scale items (4-point scale), single-choice questions, and open-ended responses.

The survey was designed to evaluate practical applicability rather than psychometric properties of the SSA-PA itself.

### 2.5. Validation

Content Validity:

Content validity was assessed by three independent experts in nursing consultation and caregiver support. The experts had between 8 and 20 years of professional experience in statutory counseling and academic teaching in nursing science. They evaluated the survey items regarding:

- Relevance,
- Clarity,
- Comprehensiveness,
- Practical applicability.

The Item-Level Content Validity Index (I-CVI) ranged from 0.67 to 1.0. Items with I-CVI = 0.67 were revised linguistically to improve clarity and reduce ambiguity. The Scale-Level Content Validity Index (S-CVI/Ave) was 0.93, indicating high overall content validity. The experts’ feedback primarily led to:

- Simplification of wording in implementation-related items,
- Clarification of response options,
- Reduction in potential redundancy.

In their written feedback, the experts particularly emphasized the practical relevance of items addressing implementation feasibility and digital usability. They recommended simplifying technical terminology in two items and clarifying response scales to avoid ambiguity. One expert suggested differentiating between structural and emotional implementation barriers, which led to minor wording revisions. Overall, the experts confirmed the survey’s comprehensiveness and relevance for evaluating practical applicability in statutory counseling contexts.

Reliability:

Internal consistency of the questionnaire was assessed using Cronbach’s alpha, a commonly applied measure of internal reliability for multi-item scales. Cronbach’s alpha was calculated for the 23 evaluative items of the survey (excluding sociodemographic variables) using standard reliability analysis procedures in IBM SPSS Statistics (Version 29). The overall reliability coefficient was α = 0.86, indicating good internal consistency according to conventional thresholds (α ≥ 0.80) reported in methodological literature. Given the exploratory design and small sample size (*n* = 13), this reliability estimate should be interpreted as preliminary.

### 2.6. Data Analysis

Quantitative analysis:

Quantitative data obtained from Likert-scale and single-choice items were analyzed descriptively using IBM SPSS Statistics Version 29 (IBM Corp., Armonk, NY, USA).

Likert-scale responses were numerically coded (1 = very useful/very important; 4 = not useful/not important). For consistency, lower mean values indicate more positive evaluations of usefulness and relevance. Descriptive statistics included means, standard deviations, as well as absolute and relative frequencies.

Item-level mean values ranged from 1.08 to 4.23, indicating variability in perceived agreement and relevance across different aspects of the SSA-PA. Standard deviations ranged from 0.28 to 2.05, suggesting that while some items showed high consensus, others reflected more heterogeneous assessments among participants.

Given the moderate sample size (*n* = 13), inferential statistical analyses were not conducted. The study was exploratory in nature and not designed to test hypotheses or detect statistically significant differences. Quantitative results therefore serve descriptive and contextual purposes within the mixed-methods framework.

Qualitative analysis:

Responses to open-ended questions were analyzed using thematic analysis following the six-phase approach described by Braun and Clarke. The analysis involved:

1. Familiarization with the data,
2. Initial coding of meaningful units,
3. Searching for themes,
4. Reviewing themes,
5. Defining and naming themes, and
6. Producing the analytical narrative.

Two researchers independently coded the qualitative material to enhance credibility and reduce interpretive bias. Coding discrepancies were discussed until consensus was reached. Themes were developed inductively to reflect participants’ perspectives on benefits, challenges, and practical implementation aspects of the SSA-PA. To enhance transparency, thematic frequencies were documented descriptively. However, frequency counts were interpreted as indicators of prominence rather than statistical weight. Illustrative quotations were selected to represent central thematic patterns and to enhance authenticity.

Integration of Quantitative and Qualitative Data

Quantitative and qualitative findings were integrated during interpretation. While descriptive statistics provided an overview of general acceptance and applicability, thematic analysis offered contextualized insights into perceived implementation barriers and support needs. This complementary approach allowed for a more comprehensive understanding of the practical relevance of the SSA-PA in statutory care counseling.

### 2.7. Consideration of Digital Competencies and Barriers

The survey explicitly included items addressing technical feasibility, digital competencies, and data protection concerns. These aspects were considered essential implementation variables, particularly given the increasing digitization of counseling services.

Findings related to digital barriers were analyzed both descriptively and thematically.

### 2.8. Reflexivity

Given that members of the research team were involved in the initial development of the SSA-PA, reflexive measures were implemented:

- Independent coding by researchers not involved in tool development.
- Team-based discussion of thematic interpretations.
- Transparent reporting of both positive and critical feedback.

Potential social desirability bias among participating care advisors cannot be excluded, particularly given the professional context and voluntary participation.

### 2.9. Ethics

The study was conducted in accordance with the Declaration of Helsinki. Written informed consent was obtained from all participants. The study protocol was approved by the responsible ethics committee. Participation was voluntary, and data were anonymized prior to analysis.

### 2.10. Limitations

Several methodological limitations must be acknowledged. First, although the sample size (*n* = 13) is larger than typical pilot studies, it remains moderate and limits generalizability. The study was not designed for inferential statistical testing or outcome evaluation.

Second, participation was voluntary, which may have introduced selection bias. Care advisors with a particular interest in innovation or digital tools may have been more likely to participate.

Third, data were based on self-reported perceptions rather than objective outcome measures. Consequently, results reflect subjective evaluations of usefulness and feasibility rather than measurable counseling effectiveness.

Finally, given the involvement of members of the research team in the development of the SSA-PA, potential confirmation bias cannot be fully excluded despite implemented reflexivity measures.

## 3. Results

### 3.1. Use and Acceptance of the Self-Assessment Tool

Overall, care advisors reported a high level of acceptance of the SSA-PA in routine counseling practice. All participants (*n* = 13) rated the tool as useful. The majority expressed clear willingness to integrate the instrument into time-limited counseling sessions, although some variability was observed regarding its practical feasibility within constrained consultation settings.

**Figure 2:**
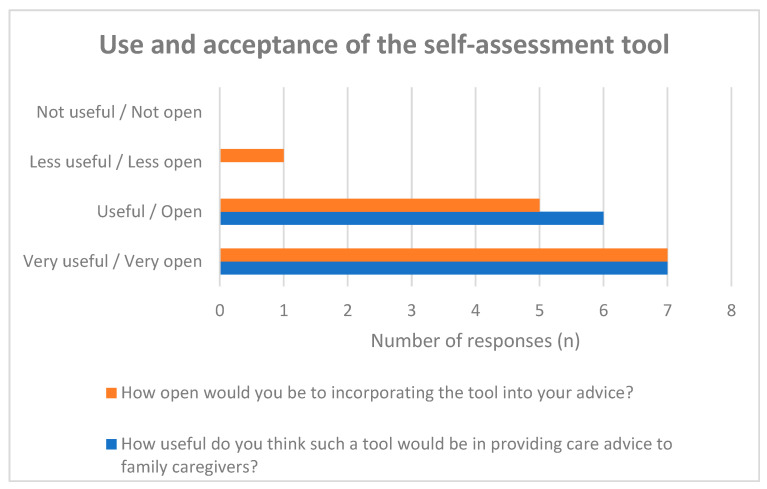
Assessment of the usefulness of the self-assessment tool and acceptance of its inclusion in counseling.

Descriptive analysis showed consistently positive ratings across usefulness items (see Figure 2). Given the exploratory design and limited sample size, these findings should be interpreted as preliminary indications of practical acceptance rather than evidence of effectiveness.

### 3.2. Importance of Integrating Self-Care Strategies

**Figure 3:**
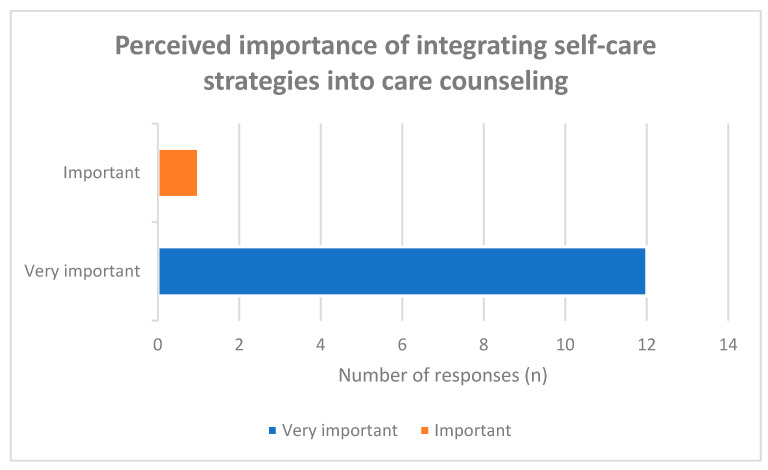
Assessment of the importance of actively integrating self-care strategies into nursing care counseling.

All respondents emphasized the importance of actively integrating self-care strategies into care counseling. Specifically, 12 of 13 participants (92%) rated integration as “very important,” while one participant (8%) rated it as “important” (Figure 3). No participant selected lower response categories.

The distribution indicates a strong consensus regarding the conceptual relevance of self-care in counseling practice. However, the item assessed general importance rather than the effectiveness of specific interventions.

### 3.3. Potential Challenges During Implementation

**Figure 4:**
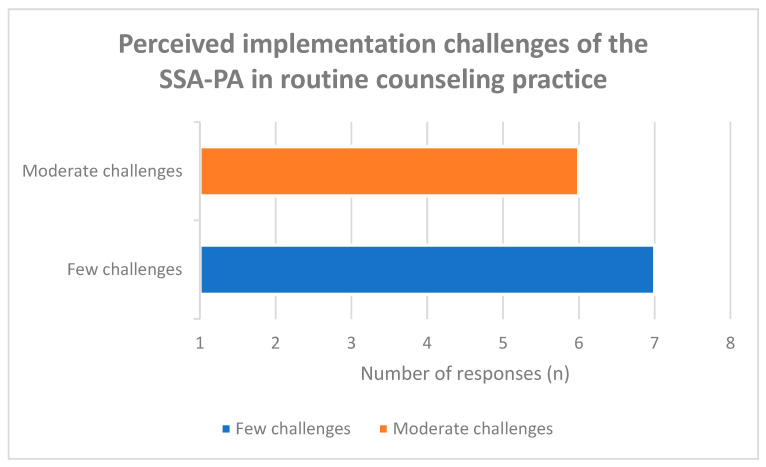
Assessment of potential challenges in implementing the new tool in training courses.

Perceived implementation challenges were predominantly rated as low to moderate. Seven participants (54%) reported few anticipated challenges, while six (46%) expected moderate challenges (Figure 4). No respondent indicated severe barriers.

**Figure 5:**
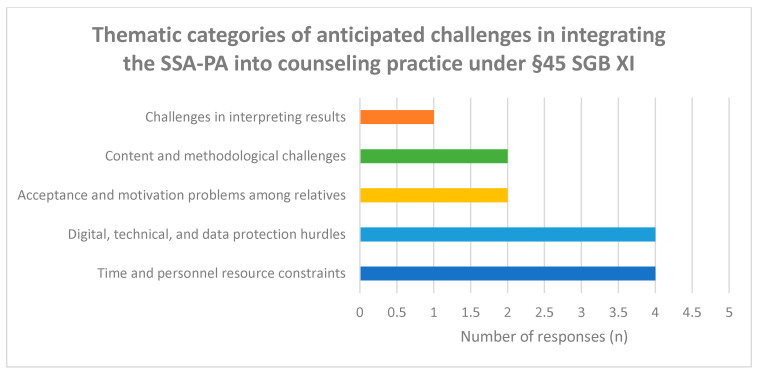
Qualitative categorization of reported challenges related to the integration of the SSA-PA into routine counseling processes.

Qualitative responses, however, revealed differentiated concerns (Figure 5). These included potential misunderstandings of questionnaire items, emotional strain among caregivers, additional time demands within consultations, and limited structural resources for follow-up. Technical barriers such as insufficient digital competencies, lack of equipment, and data protection uncertainties were also mentioned.

Thus, while the quantitative ratings suggest manageable implementation barriers, qualitative data indicate practical considerations that may influence routine integration.

### 3.4. Assessment of Stress Using the Tool

**Figure 6:**
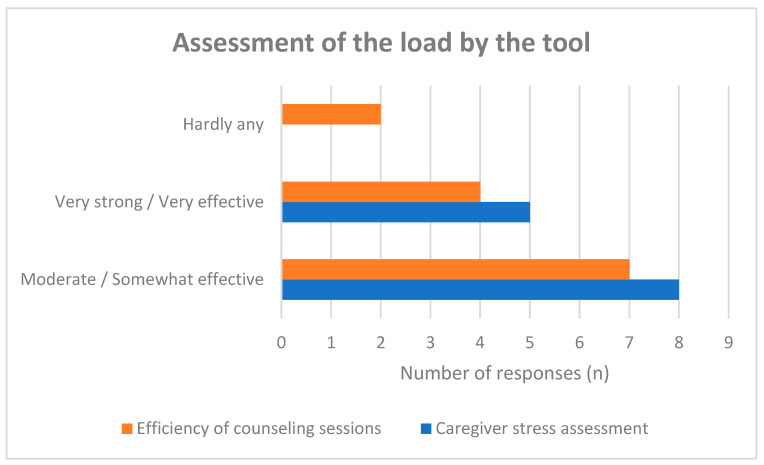
Assessment of the load by the tool.

Most care advisors perceived the SSA-PA as supportive for helping caregivers reflect on and assess their own stress levels. Eight participants rated the tool’s contribution to stress assessment as “moderate/somewhat effective,” and five as “very strong/very effective” (Figure 6).

In contrast, assessments of potential efficiency gains in counseling were more heterogeneous. While several respondents perceived improvements through structured preparation and self-reflection, others reported only limited impact on consultation duration or workflow.

These findings suggest that the perceived value of the SSA-PA may lie more strongly in reflective and diagnostic functions than in measurable efficiency gains.

### 3.5. Added Value and Benefits of the SSA-PA

**Figure 7:**
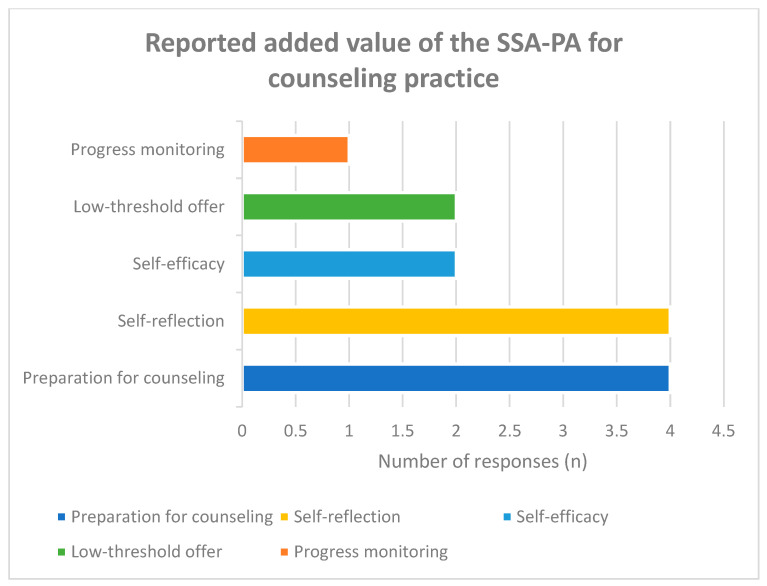
Perceived benefits and added value of using the self-assessment tool.

Participants identified multiple dimensions of added value associated with the SSA-PA (Figure 7). The most frequently reported benefits included improved preparation for counseling (31%), enhanced self-reflection among caregivers (31%), and increased perceived self-efficacy (15%). Additional advantages included low-threshold access (15%) and the potential for progress monitoring (8%).

**Figure 8:**
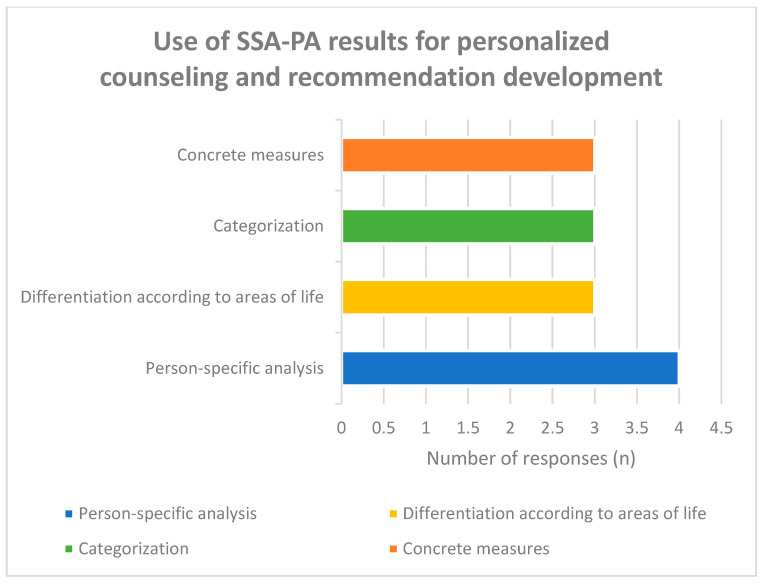
Assessment of the use of tool results to analyze the individual support needs of family caregivers and to derive personalized recommendations.

Regarding the use of tool results for personalized counseling (Figure 8), responses were relatively evenly distributed. Four participants (31%) emphasized person-specific analysis, three (23%) highlighted differentiation according to life domains, three (23%) referred to categorization of burden types, and three (23%) emphasized derivation of concrete measures.

This distribution indicates that respondents perceived the SSA-PA not merely as a screening instrument but as a structured basis for individualized counseling and recommendation development.

### 3.6. Necessary Adjustments, Additional Functions, and User Feedback

The qualitative findings related to necessary adjustments, additional functions, and feedback requirements for further development of the SSA-PA are summarized in Table 3. The following sections describe and contextualize these findings.

#### 3.6.1. Necessary Adjustments to Enhance Practical Applicability

Care advisors identified several necessary adjustments to improve the practical applicability of the SSA-PA within care counseling pursuant to §45 SGB XI. The most frequently mentioned adjustment concerned the need for automated evaluation and structured result processing, allowing for more efficient analysis and integration into counseling procedures. Closely related to this, respondents emphasized the importance of generating concrete recommendations for action and references to appropriate support services to facilitate targeted follow-up.

Further adjustments referred to the implementation of history and documentation functions to ensure continuity and traceability within the counseling process. In addition, participants highlighted the need to enhance user-friendliness and accessibility, as well as options for customization and stronger system support to better align the tool with existing counseling structures and practical requirements.

#### 3.6.2. Suggested Additional Functions

With regard to further development, care advisors outlined several options to better align the SSA-PA with the complex and situation-specific needs of family caregivers in counseling pursuant to §45 SGB XI. Most prominently, respondents emphasized the importance of increasing the tool’s flexibility, allowing for stronger differentiation and adaptation to individual caregiving constellations and burden profiles.

Moreover, participants proposed reinforcing the resource- and strength-based components of the instrument in order to more systematically incorporate caregivers’ existing capacities. Additional suggestions included more advanced evaluation and visualization features to enhance clarity and comprehensibility of results, as well as the integration of elements that explicitly address self-care. One respondent reported that no further modifications were considered necessary.

#### 3.6.3. Feedback from Family Caregivers as a Development Resource

Care advisors underscored the relevance of systematically incorporating feedback from family caregivers to enable ongoing refinement of the SSA-PA. Such feedback was considered essential to ensure that the tool remains aligned with caregivers’ needs and the practical requirements of counseling under §45 SGB XI.

In particular, respondents emphasized the importance of gathering feedback on the completeness of the content and potential areas for further development, as well as on the perceived practical usefulness and impact of the tool within the counseling process. Additionally, assessments of comprehensibility and user-friendliness were regarded as relevant, alongside feedback on the emotional strain and time pressure associated with completing the instrument. Overall, participants highlighted the value of establishing a continuous and structured feedback process to support sustainable adaptation and quality assurance.

### 3.7. Long-Term Use of the Self-Assessment Tool Beyond Counseling

The qualitative findings regarding the use of the SSA-PA beyond individual counseling sessions are summarized in Table 4. Two main thematic areas were identified in the open-ended survey responses.

#### 3.7.1. Long-Term Support Through the Tool

Care advisors reported that the SSA-PA may contribute to sustained self-reflection and self-care beyond the immediate counseling context. Qualitative responses emphasized the establishment of regular repetition and structured self-assessment as a key mechanism for supporting caregivers over time. Recurrent use was described as enabling caregivers to reflect on changes in their burden and personal resources.

In addition, respondents highlighted the importance of facilitating progress tracking and long-term documentation to make developments visible across assessment points. The provision of specific recommendations for self-care and concrete courses of action was considered central to promoting active coping beyond the counseling session. Furthermore, participants referred to the integration of automated warning or support systems to signal critical changes, as well as ensuring low-threshold access to foster continuous engagement and reflection.

#### 3.7.2. Use of the Tool for Regular Reflection

Care advisors further described the SSA-PA as a structured instrument for ongoing reflection and monitoring within caregiving arrangements. The most frequently mentioned approach involved establishing regular and event-related repetition to reassess the caregiving situation at defined intervals or in response to significant changes.

Respondents also emphasized the need for systematic documentation and progress tracking to enable transparent comparison over time. To ensure sustainable implementation, organizational and technical support structures were considered necessary, for example, through integration into existing counseling processes. One respondent expressed general consent to regular use of the tool as a basis for continuous reflection.

### 3.8. Integration of the Self-Assessment Tool into the Counseling Process

The qualitative findings related to the integration of the SSA-PA into care counseling processes are summarized in Table 5. Five main thematic areas were identified in the open-ended survey responses. 

#### 3.8.1. Steps for Integration into the Counseling Process

Care advisors described several approaches for integrating the SSA-PA into counseling practice in accordance with §45 SGB XI. Qualitative responses most frequently referred to the use of the tool as a preparatory instrument prior to the appointment in order to obtain relevant information in advance and structure the upcoming consultation.

In addition, respondents reported involving the tool directly during the consultation to guide discussion and clarify specific issues. Some participants also described its use in the further course of counseling to reassess developments or accompany ongoing processes. Structural anchoring within existing organizational procedures was considered important to ensure routine implementation. One respondent indicated that no additional integration steps were necessary.

#### 3.8.2. Time of Use Within the Counseling Process

Care advisors predominantly identified the beginning of the counseling process as the optimal time for applying the SSA-PA. Most qualitative responses emphasized its use at the initial stage to gain a comprehensive overview of the caregiving situation and to identify relevant topics at an early point.

A smaller number of responses referred to the possibility of using the tool in the middle of the counseling process, depending on situational needs and the progression of the consultation.

#### 3.8.3. Personalization of Counseling Based on Tool Results

Qualitative responses indicated that the results of the SSA-PA can serve as a foundation for targeted problem analysis and prioritization within individualized counseling sessions. Care advisors described preparing and presenting results in a structured manner during the consultation to facilitate clarity and focus.

Furthermore, respondents emphasized the value of joint reflection and prioritization together with family caregivers. Consideration of progression and developmental aspects over time was also mentioned, alongside the use of results to support concrete measures and structured goal planning within the counseling process.

#### 3.8.4. Systematic Evaluation of Assessment Results

Care advisors outlined several methods for the systematic evaluation of SSA-PA results and the derivation of recommendations. Frequently mentioned approaches included AI- and algorithm-based evaluation models to support efficient and consistent processing of data.

In addition, respondents referred to standardized analysis procedures and structured point or scoring systems to ensure comparability and transparency. Automated processing of results and the possibility of integrating historical data and conducting multiple data analyses over time were also highlighted as relevant components of a systematic evaluation framework.

#### 3.8.5. Training Requirements for Care Advisors

Care advisors expressed varying perspectives regarding training requirements for the effective use of the SSA-PA. A substantial proportion of respondents indicated that no or only minimal training would be required, particularly if evaluation and processing procedures are largely automated.

Others noted that training needs may depend on the degree of automation implemented within the tool. Additional competencies mentioned included technical and methodological skills, as well as conversational skills and awareness for addressing sensitive topics. Some respondents also referred to the relevance of further qualification measures and professional networking to support sustainable implementation.

## 4. Discussion

### 4.1. Interpretation of Key Findings in Relation to Counseling Practice

This study explored the perceived usefulness and applicability of the Self-Assessment Tool for Family Caregivers (SSA-PA) from the perspective of care advisors working under §45 SGB XI. Based on responses from 13 participants, the findings indicate a consistently high level of perceived acceptance and conceptual relevance of the tool in routine counseling practice.

All respondents rated the SSA-PA as useful, and most expressed willingness to integrate it into time-limited preventive counseling sessions. At the same time, variability in perceived efficiency gains suggests that the tool is not primarily understood as a time-saving intervention, but rather as a structuring and reflective support instrument. This interpretation aligns with the finding that advisors perceived stronger contributions to stress assessment and prioritization than to measurable counseling efficiency.

Importantly, these findings complement earlier results obtained from family caregivers themselves [26] by adding the professional perspective of care advisors who apply the instrument within statutory preventive counseling settings. Similar to prior caregiver feedback, the present results indicate that the differentiation across life domains supports articulation of burden and facilitates structured discussion.

The strong consensus regarding the importance of integrating self-care strategies into counseling reflects broader international evidence emphasizing preventive and reflective approaches in caregiver support [10,11,12,13,14,15]. However, it must be emphasized that the present study assessed perceived usefulness and professional acceptance rather than objective effectiveness. Thus, the results should be interpreted as preliminary, context-specific evidence rather than proof of measurable impact on caregiver burden or counseling outcomes.

Comparable findings have been described in international studies on structured reflection tools in caregiver counseling, where the primary benefit lies in improving communication, awareness, and prioritization rather than efficiency alone [16,17,18,21]. The added value of the SSA-PA therefore appears to lie primarily in facilitating dialogue and shared understanding within counseling processes.

### 4.2. Use of the SSA-PA as a Reflective and Longitudinal Support Instrument

Beyond its immediate use within single counseling sessions, care advisors described the SSA-PA as a potential instrument for ongoing reflection and longitudinal monitoring of caregiver burden. Qualitative responses emphasized repeated application, structured documentation, and progress tracking as mechanisms that could support sustained self-reflection.

These perceptions are consistent with international research highlighting the importance of continuous monitoring and reflective practices in long-term caregiving trajectories [9,16,25]. Advisors proposed integrating automated evaluation systems, visualization features, and structured feedback processes to strengthen longitudinal usability.

At the same time, the present study did not examine actual repeated use or longitudinal effects. The perceived potential for long-term support therefore remains hypothetical and requires empirical validation. Future studies should investigate whether structured reassessment using the SSA-PA contributes to earlier detection of burden escalation or improved coping strategies over time.

### 4.3. Integration into Counseling Processes: Practical Implications

The findings demonstrate that the SSA-PA is perceived as flexible regarding timing and integration within counseling workflows. The majority of respondents identified the beginning of the counseling process as the optimal point of application, as it enables early identification of relevant topics and structured prioritization.

This observation is in line with international caregiver counseling models that employ structured screening or reflection tools to personalize support and enhance caregiver engagement [16,17,21]. The relatively even distribution of responses concerning personalization strategies—such as differentiation by life domains, burden categories, or derivation of concrete measures—indicates that the tool allows individualized counseling without imposing rigid procedural requirements.

However, successful integration appears dependent on structural and organizational conditions. Advisors emphasized the need for automated evaluation, structured visualization of results, and compatibility with existing documentation systems. These system-level requirements reflect broader implementation challenges described in caregiver support research, where technological and infrastructural factors significantly influence adoption [1,2,3,4,5].

Training requirements were perceived as generally low, particularly in contexts with automated processing. Nevertheless, some participants highlighted the importance of technical competencies and conversational skills when addressing emotionally sensitive topics. These findings underline that structured assessment tools may enhance counseling processes but do not replace professional judgment or relational competence.

### 4.4. Challenges, Biases, and Methodological Considerations

Although quantitative ratings suggested predominantly low-to-moderate implementation barriers, qualitative responses revealed more differentiated concerns. These included potential emotional strain associated with confronting caregiver burden, misunderstandings of questionnaire items, time constraints within consultations, digital literacy barriers, and data protection considerations.

The combination of positive overall acceptance with nuanced qualitative feedback illustrates the importance of mixed-methods designs in implementation research. While the SSA-PA appears broadly acceptable among care advisors, contextual factors and individual caregiver characteristics may influence its practical usability.

Several methodological limitations must be acknowledged. Despite the increased sample size (*n* = 13), the study remains exploratory and context-specific. Statistical generalization is not possible, and findings should be interpreted cautiously. Moreover, the evaluation focused exclusively on perceived usefulness and anticipated integration rather than measurable outcomes such as reduction in caregiver burden, improvement in counseling quality, or health-related effects.

Social desirability bias cannot be excluded. Participants may have evaluated the tool favorably due to professional norms or openness toward innovation. Although anonymity was ensured, such influences must be considered when interpreting consistently positive ratings.

Finally, the study focused solely on the perspective of care advisors. Although caregiver feedback has been examined previously [26], integrating both perspectives within a unified research design would provide a more comprehensive assessment of emotional impact, usability, and practical relevance.

### 4.5. International Relevance and Transferability

The SSA-PA was developed and evaluated within the German statutory framework of §45 SGB XI. This legal and organizational embedding limits direct transferability to other national contexts.

Nevertheless, several core mechanisms identified in this study—structured self-reflection, differentiation across life domains, facilitation of dialogue, and personalization of recommendations—address needs that are consistently described in international caregiver research [1,2,3,4,5,16,22,28,29,30]. These structural functions are not inherently bound to the German legal framework and may therefore be adaptable to other healthcare or community-based support systems.

However, transferability should not be assumed without careful adaptation. Differences in counseling structures, reimbursement systems, cultural norms, and family role expectations may influence both interpretation of items and integration into practice. Cross-national pilot studies and culturally sensitive validation processes would therefore be necessary before broader implementation can be recommended.

### 4.6. Limitations and Directions for Future Research

Several limitations must be considered when interpreting the present findings. Although the sample size was expanded to 13 participants, the study remains exploratory and context-specific. The descriptive design does not allow causal inference or statistical generalization.

The evaluation focused on perceived usefulness, acceptance, and implementation perspectives. No objective outcome measures were collected, and no conclusions can be drawn regarding the effectiveness of the SSA-PA in reducing caregiver burden or improving counseling outcomes.

Future research should include larger and more diverse samples, integrate caregiver-reported outcome measures, and examine longitudinal effects of repeated tool use. Controlled implementation studies could clarify whether structured self-assessment contributes to earlier burden recognition or improved counseling quality. Furthermore, international pilot projects would be valuable to explore cultural adaptation and context-specific feasibility.

## 5. Conclusions

This exploratory study provides initial empirical insights into the perceived applicability and professional acceptance of the SSA-PA from the perspective of care advisors working under §45 SGB XI (*n* = 13). The findings suggest that the tool is regarded as a useful and flexible instrument for structuring counseling sessions, supporting caregiver self-reflection, and facilitating the systematic identification of burden across life domains.

At the same time, the results indicate areas for further refinement, particularly regarding clarity of response options, low-threshold usability, automated evaluation features, and practical feedback mechanisms that translate assessment results into actionable counseling strategies. The mixed-methods design also revealed that, despite generally positive ratings, contextual and structural factors may influence implementation in routine practice.

Given the exploratory design and context-specific sample, the findings should be interpreted cautiously. The study assessed perceived usefulness rather than objective effectiveness, and no conclusions can be drawn regarding measurable outcomes for caregivers. Future research should therefore include larger samples, caregiver-reported outcome measures, and longitudinal or controlled implementation designs to evaluate potential effects on counseling quality and caregiver well-being.

Although the SSA-PA was developed within the German statutory framework, its underlying conceptual focus on structured self-reflection and preventive support corresponds to internationally recognized challenges in family caregiving. With appropriate cultural and organizational adaptation, the instrument may hold relevance beyond the national context. However, cross-national validation and implementation studies are required before broader applicability can be assumed.

## Figures and Tables

**Table 1:** Sociodemographic data.

Participant	Age	Gender	Educational Qualification	Consulting Experience (§45 SGB XI)
Person 1	Under 30 years old	Female	University degree	1–3 years
Person 2	50–59 years old	Not specified	University degree	1–3 years
Person 3	Under 30 years old	Male	University degree	More than 3 years
Person 4	30–39 years old	Female	University degree	More than 3 years
Person 5	40–49 years old	Female	Vocational training	More than 3 years
Person 6	Under 30 years old	Female	University degree	Less than 6 months
Person 7	Under 30 years old	Female	University degree	1–3 years
Person 8	30–39 years old	Female	University degree	More than 3 years
Person 9	40–49 years old	Male	University degree	1–3 years
Person 10	50–59 years old	Female	Vocational training	More than 3 years
Person 11	Under 30 years old	Not specified	University degree	Less than 6 months
Person 12	30–39 years old	Female	University degree	More than 3 years
Person 13	40–49 years old	Male	University degree	1–3 years

**Table 2:** Overview of item categories.

Category	Content Focus	Number of Questions
1	Usefulness & relevance	5
2	Acceptance	1
3	Challenges	2
4	Further development	2
5	Sustainability	2
6	Integration into the process	2
7	Use of results	3
8	Training needs	1
9	Feedback & evaluation	1
10	Sociodemographics	4

**Table 3:** Core categories and frequency of mentions regarding adjustments, additional functions, and feedback for the self-assessment tool in care counseling pursuant to Section 45 of SGB XI.

Question	Core Categories	Number of Mentions
1. What adjustments would be necessary to make the tool for care advice in accordance with Section 45 of SGB XI even more relevant in practice? Are there any specific contents or functions that should be added?	Automated evaluation & result processing	4
	Recommendations for action & support services	3
	History & documentation functions	2
	User-friendliness & accessibility	2
	Customization & System Support	2
2. What adjustments or additional functions could be made to tailor the tool even better to the specific needs and burdens of family caregivers in counseling pursuant to Section 45 of SGB XI?	Greater individualization and adaptation to specific situations	5
	Advanced evaluation and visualization	2
	Expand resource and strength orientation	3
	Integrate support for self-care	2
	No further adjustments or additional functions required	1
3. What kind of feedback from family caregivers would be useful in order to continuously adapt the tool to their needs and the requirements of care counseling in accordance with §45 SGB XI?	Feedback on the completeness of the content and further development of the tool	4
	Assessments of the practical usefulness and impact of the tool	3
	Feedback on comprehensibility and user-friendliness	2
	The emotional and time pressure involved in filling out the form	2
	A continuous and structured feedback process	2

**Table 4:** Core categories and frequency of mentions to support self-reflection and self-care through the self-assessment tool.

Question	Core Categories	Number of Mentions
1. How could the tool help to support family caregivers in self-reflection and self-care in the long term after counseling?	Establish regular repetition and self-assessment	3
	Enable progress tracking and long-term documentation	2
	Provide specific recommendations for self-care and action	3
	Integrate automated warning and support systems	2
	Ensure low-threshold use and promotion of self-reflection	2
2. How could the tool be used as a regular reflection tool to track progress over time?	Establish regular and event-related repetition	5
	Enable systematic documentation and progress tracking	4
	Ensure organizational and technical support	3
	Basic consent to regular use	1

**Table 5:** Core categories and frequency of mentions regarding the use and integration of the self-assessment tool in counseling processes.

Question	Core Categories	Number of Mentions
1. Steps/methods for integrating the tool into the consulting process	Use as a preparatory tool before the appointment	4
	Involvement during the consultation	3
	Use in the further course of consultation	2
	Structural anchoring in the system	3
	No additional integration needs identified	1
2. Optimal timing for use in the consulting process	At the beginning	11
	In the middle	2
3. Integration of the results of the self-assessment tool into individualized counseling sessions	Results for targeted problem analysis and prioritization	4
	Prepare and present results in a structured manner during the consultation	3
	Encourage joint reflection and prioritization with relatives	2
	Consider aspects of progression and development	2
	Support concrete measures and target planning	2
4. Methods for systematic evaluation and derivation of recommendations	AI and algorithm-based evaluation	4
	Point systems and structured assessment	2
	Standardized analysis methods	3
	Automated processing of results	2
	History and multiple data analysis	2
5. Training requirements for consultants for efficient use of the tool	No or little training required	5
	Depending on the degree of automation of the tool	1
	Technical and methodological skills	3
	Conversation skills and awareness raising	2
	Further qualification and networking	2

## Data Availability

The raw data supporting the conclusions of this article will be made available by the authors on request.
